# Cytotoxicity and molecular docking analysis of phytochemicals from *Vallisneria spiralis* with protein target 3CZH in breast cancer management

**DOI:** 10.6026/9732063002002062

**Published:** 2024-12-31

**Authors:** Ravindra Waykar, Srinivasakumar Kumarapillai

**Affiliations:** 1 Department of Pharmacy, Lincoln University College, Wisma Lincoln, No. 12-18, Jalan SS 6/12, 47301 Petaling Jaya, Selangor Darul Ehsan, Malaysia

**Keywords:** *Vallisneria spiralis Linnaeus*, breast cancer, Silver nanoparticles (AgNPs), Iron oxide nanoparticles (IONPs), *In Silico* analysis, *in vitro* analysis

## Abstract

The anticancer potential of *Vallisneria spiralis Linnaeus* (*Vallisneria spiralis L*.) for human breast cancer management is of interest. *In vitro* shows that increasing concentrations of *Vallisneria spiralis* silver nanoparticles (AgNPs) and iron oxide nanoparticles (IONPs) resulted in greater cytotoxicity against MCF-7 breast cancer cells. The antioxidant potential of *Vallisneria spiralis L*. was assessed using 2-diphenyl-1-picryl-hydroxyl (DPPH) radical scavenging assay. Further molecular docking analysis of phytocompounds from *Vallisneria spiralis L*. with the target protein (PDB ID: 3CZH) shows optimal binding features. Thus, cytotoxicity analysis of *Vallisneria spiralis* for breast cancer with molecular docking of its phytochemicals and a target protein 3CZH is reported for further consideration.

## Background:

The leading cause of cancer-related mortality worldwide is breast cancer, the most common kind of malignant cancer [1]. In 2020, the number of breast cancer diagnoses reached approximately two million worldwide [2]. Surgery is still the primary treatment strategy, although it is frequently used in conjunction with other techniques such immunotherapy, endocrine therapy, radiation, chemotherapy and targeted therapy [3]. A promising alternative involves plant-derived secondary metabolites, which provide readily accessible and potent natural compounds containing various bioactive elements with anti-cancer properties [4]. Forecasts suggest that by 2040, the annual cancer-related fatalities will reach approximately 16 million [5]. Conventional cancer treatments, such as chemotherapy and radiotherapy, frequently lead to adverse effects, including physical effects such as fatigue, nausea, anemia and oxygen deprivation, as well as psychological consequences such as stress [6]. The development of new anticancer drugs with reduced toxicity and enhanced therapeutic efficacy has become essential [7]. A significant trend has emerged, moving away from synthetically produced pharmaceuticals towards naturally derived substances, with numerous scientific studies exploring the cancer-fighting potential of plant-based compounds [8].

The Notch signaling system, a fundamental biological mechanism conserved across numerous organisms, is vital for cellular processes including proliferation, division and fate determination [9]. This system exerts a significant influence on mammary development, haematopoiesis and colorectal epithelial maturation [10]. Dysregulation of the Notch signaling cascade is implicated in malignant growth, other epithelial cancers and chronic intestinal inflammation [11]. Depending on the type of cancer, Notch signaling pathways have varied effects on tumor growth [12]. These effects include angiogenesis, differentiation, cell cycle progression, cellular metabolism and immunological functions [13]. A recent analysis underscored the significance of Notch receptors in the tumorigenic activity of Notch signaling pathways across multiple cancer types, including lung adenocarcinoma, cervical cancer, haematological malignancies, breast cancer and ovarian cancer [14]. Ligand binding and activation are critically dependent on the extracellular portion, which is characterized by multiple repeating units, similar to epidermal growth factor (EGF) [15]. The activation of Notch receptors may be affected by phytocompounds through their interactions with EGF-like repeats [16]. The abnormal functioning of Notch signaling in cancer treatment promotes the survival and growth of cancer cells, making the targeting of specific regions, such as the extracellular domain responsible for ligand binding, a potential approach for therapeutic intervention [17]. Therefore, it is of interest develop and evaluate a drug delivery system using nanoparticles derived from *Vallisneria spiralis L*., specifically for breast cancer treatment.

## Materials and Methods:

Analytical-grade chemicals and reagents were used exclusively in the experimental procedures. Molecular Phylogenetic Analysis of the specimen was performed as described previously [18]. The phytochemicals of *Vallisneria spiralis L*. were extracted and identified, as previously described [19]. Synthesis and Characterization of *Vallisneria spiralis L*. silver nanoparticles and Iron nanoparticles were conducted as described previously [20].

## Antioxidant assay:

The antioxidant potential of *Vallisneria spiralis L*. was assessed using a 2-diphenyl-1-picryl-hydroxyl (DPPH) solution [21]. Stock solutions were prepared by dissolving *Vallisneria spiralis L*. extracts, ascorbic acid at a concentration of 5 mg/mL, in 95% methanol and DPPH at 0.004% w/v. Extracts of *Vallisneria spiralis L*. were diluted to various concentrations ranging from 50 to 250 µg/mL using standard reagents through serial dilution techniques. The DPPH test was performed by combining 0.1 mL of each sample with 3 mL of freshly prepared DPPH solution. This mixture was then stored in the dark for 30 min. Equal parts DPPH and 0.1 millilitres of methanol were added to prepare a control sample. Following incubation, absorbance at 517 nm was measured using a spectrophotometer. Free radical scavenging activity was indicated by reduced absorbance in the sample.

The percentage of inhibitory activity was calculated using the following formula:

Free radicals inhibition (%) = [A control - A sample] / A control x 100

## *In-vitro* cytotoxicity assay:

The human breast cancer (BCa) cells used in this study were MCF-7 cells from the National Center for Cell Science in the Pune district of the Maharashtra state of India. The cells were cultured in high-glucose Dulbecco's Modified Eagle Medium, which was supplemented with one per cent antibiotic antimycotic solution and ten percent fetal bovine serum. The culture conditions were maintained at 37°C in a CO2 incubator with an atmosphere containing 5% CO2 and 18-20% O2. MCF-7 breast cancer cells were cultured to determine the percentage of cell viability and half-maximal inhibitory concentration values, *i.e.*, IC50. Cultured cells were divided into four groups according to the treatment administered. The initial cohort served as the negative control and received no treatment. Experimental group number two and three received different concentrations of Silver nanoparticles (AgNPs) and Iron Oxide nanoparticles (IONPs) derived from *Vallisneria spiralis L*. Camptothecin was administered to the fourth group as the standard control treatment. A standardized method was utilized to evaluate the toxic effects on cells caused by extracts obtained from *Vallisneria spiralis L*. [22]. The MTT experiment involved seeding MCF-7 BCa cells in a 96-well plate, followed by treatment with varying dosages of AgNPs, IONPs, Camptothecin and the untreated cells were cultured alone on medium and incubated for aduration of twenty-four hours. Excess medium was removed from all cells by washing with a phosphate buffered saline solution. Phosphate-buffered saline solution containing the MTT reagent was added to each well and incubated for 3 h at 37 °C. The MTT dye was removed followed by the addition of 100% Dimethyl sulfoxide. The absorbance was measured at 570 nm using an ELISA plate reader. All experimental trials were replicated three times. The IC50 concentration demonstrated 50% inhibition of c ancer cells, whereas an assessment of normal cells was conducted using the nonlinear regression technique. Morphological changes resulting from the inhibitory effects of different doses of test compounds were detected and analyzed in MCF-7 BCa cells using the Cell Imaging Station. These changes were then compared with untreated cells, which were used as the control group.

The percentage of cell viability was calculated using the following formula:

% Cell viability = [mean absorbance of treated cells / mean bsorbance of untreated cells] x 100

## Trypan blue exclusion assay:

The percentage of adherent MCF-7 BCa cells was determined using a trypan blue solution. 96-wells plate was used to grow the cells with varying concentrations (6.25-200 µg/mL) of IONPs and AgNPs of *Vallisneria spiralis L*. and Camptothecin as a standard treatment and untreated cells incubated for one day, as described in the MTT assay. The cell suspension to be evaluated is subjected to centrifugation for duration of 5 min. The pellet was reconstituted in 1 mL solution of phosphate buffered saline solution after removing the supernatant. Trypan blue dye (0.4%) was then added to the cell suspension. The solution was maintained at ambient temperature for 3 min. The hemocytometer was positioned on the microscope and a few drops of the mixture were placed onto it. A hemocyto meter was used to count the number of unstained viable and stained nonviable cells [23].

The calculation for determining the % viable cells was performed using the below equation:

% Cell viability = [total number of viable cells per mL of aliquot / total number of cells per mL of aliquot] x 100

## *In Silico* study:

## The structures of PDB ID:

3CZH for the anticancer targets was acquired from the RCSB PDB. The process of protein preparation includes eliminating water molecules. CASP server was used to find binding sites in the target proteins [24]. The Auto Dock Vina program was used to record the resulting specifications in a config.txt file [25]. Subsequently, the ligand that had undergone co-crystallization was extracted from the protein and preserved in pdbqt format. The structure of *Vallisneria spiralis L*. phyto compounds were obtained from the PubChem database in SDF format and subsequently converted into a PDB format utilizing the BIOVIA Discovery Studio visualizer for in-silico evaluation [26]. The preparation of the ligand was conducted by separate uploads into the Auto Dock Vina program. The identification of therapeutically significant candidates to serve as an effective and safe medicine relies on the analysis of pharmacokinetics and physicochemical attributes. The analysis of these traits utilizing *In-vitro* and *In-vivo* approaches proved to be both time-consuming and costly [27]. Consequently, an *In Silico* method was utilized for screening drug like compounds and toxicity analysis [28, 29- 30]. The Auto Dock Vina program was utilized for molecular docking and proteins and ligands converted into the PDBQT format, energy was reduced and found the active site [31]. The docking approach involves treating macromolecules as inflexible entities while allowing the ligands to exhibit flexibility in order to generate various conformations. Stable interactions between the ligand and the selected macromolecule, characterized by an RMSD value less than 1 Å, were used to determine the lowest favorable binding energy [32].

## Results & Discussion:

## *In-vitro* antioxidant scavenging activity:

**Figure 1:**
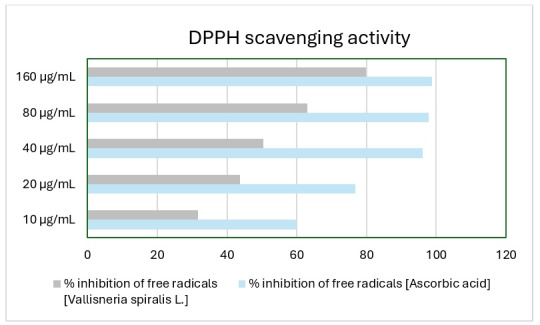
*Vallisneria spiralis L*. DPPH radical scavenging assay

An *In-vitro* model was developed to forecast the scavenging potential of methanolic extracts derived from *Vallisneria spiralis L*., as well as a reference reagent known as ascorbic acid. The antioxidative ability of *Vallisneria spiralis L*. was evaluated. Figure 1 presents the percentage of inhibition observed in the scavenging of free radicals.

## *In-vitro* cytotoxicity assay:

**Figure 2:**
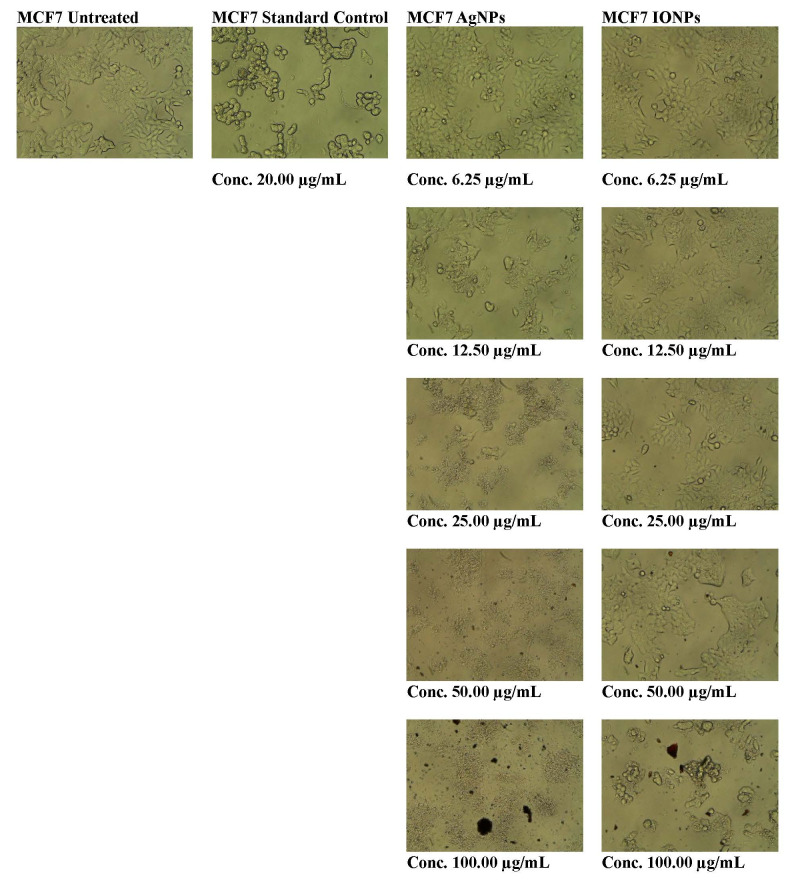
Morphology of untreated human breast cancer cells (MCF7), MCF-7 treated with standard control, *i.e.*, Camptothecin, MCF-7 treated with *Vallisneria spiralis* silver nanoparticles (AgNPs) and MCF-7 treated with *Vallisneria spiralis* iron oxide nanoparticles (IONPs).

The antiproliferative effects of *Vallisneria spiralis L*. extracts of AgNPs and IONPs (6.25-100 µg/mL) on MCF-7 BCa cell lines were determined using an MTT assay. The findings demonstrated that increasing the concentration of *Vallisneria spiralis* AgNPs and IONPs resulted in a reduction in the number of viable cells owing to the induction of greater cytotoxicity compared to untreated cells. Among the tested nanoparticles, *Vallisneria spiralis* AgNPs exhibited the highest toxicity towards cancer cell lines. These nano-particles demonstrated IC50 values of 18.26µg/mL against MCF-7 BCa cells. In comparison, *Vallisneria spiralis* IONPs showed IC50 values of 42.22µg/mL when tested against the same MCF-7 BCa cells (Figure 2).

## Trypan blue exclusion assay:

**Figure 3:**
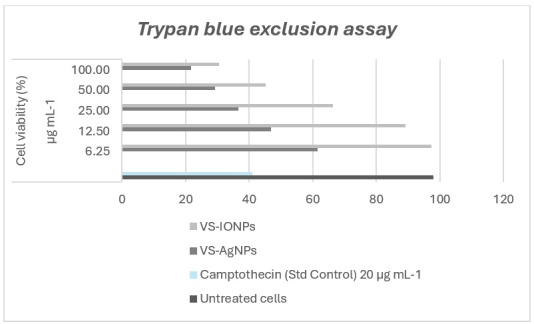
Trypan blue exclusion assay

Trypan blue exclusion assay was employed to evaluate the growth inhibition effects on MCF-7 cells at various concentrations (6.25, 12.5, 25, 50 and 100 µg/mL) of the test sample. The results showed that higher concentrations of the test sample corresponded to a greater inhibition of cell growth. Each in vitro experiment was conducted three times and the results are presented as the mean value ± standard deviation (Figure 3).

## Molecular docking analysis:

**Figure 4:**
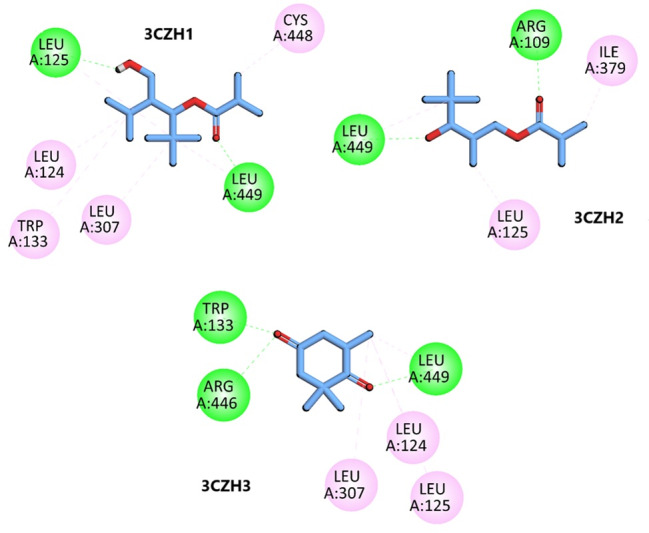
2D interaction of 3CZH and 3CZH-complex

The anti-breast cancer potential of *Vallisneria spiralis* was investigated thru docking with target protein (PDB ID = 3CZH). The predicted anticancer activity of *Vallisneria spiralis L*. phytocompounds are shown in Table 1. The phytocompounds found in *Vallisneria spiralis L*. exhibited higher Pa values compared to Pi, suggesting a greater potential for iological activity in breast cancer inhibition rather than inactivity. This finding indicates an increased likelihood that the phytocompounds from *Vallisneria spiralis L*. possess anti-breast cancer properties. Table 2 and Table 3 show the *in-silico* analyses. Compound 1 interacts with the anticancer target protein (PDB ID = 3CZH) through hydrogen bonding with key residues, including Leu125 and Leu449 and through pi-alkyl interactions with Leu124, Trp133, Leu307 and Cys448 (3CZH1, Figure 4). Compound 2 shows hydrogen bonding interactions with important residues Arg109 and Leu449 and pi-alkyl interactions with Leu125 and Ile379 in the active site of the target protein (3CZH2, Figure 4). Compound 3 exhibits conventional hydrogen bonding interactions with Trp133, Arg446 and Leu449 and pi-alkyl interactions with Leu125, Leu124 and Leu307 at the receptor site of the protein 3CZH (3CZH3, Figure 4). The binding energies between the protein (3CZH) and its ligands in the molecular docking experiments are documented in Table 4.

## *In-vitro* cytotoxicity assay:

**Figure 5:**
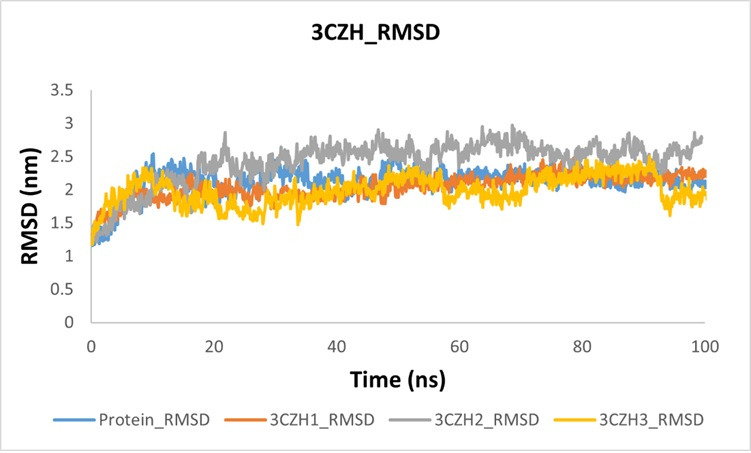
RMSD graph of 3CZH and 3CZH-complex

**Figure 6:**
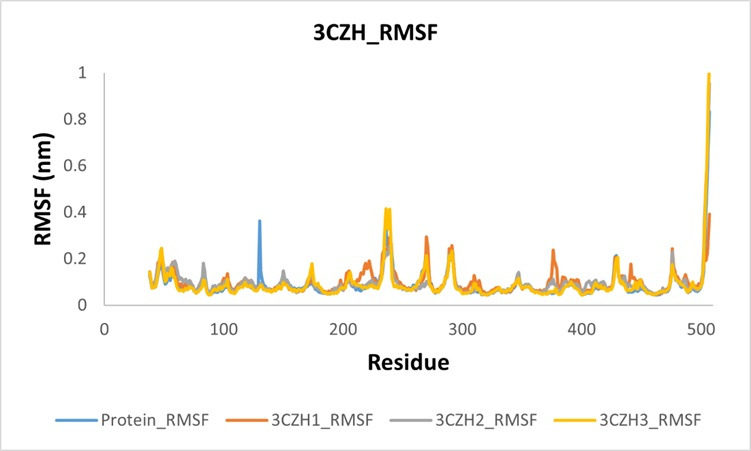
The RMSF graph of 3CZH and 3CZH-complex

**Figure 7:**
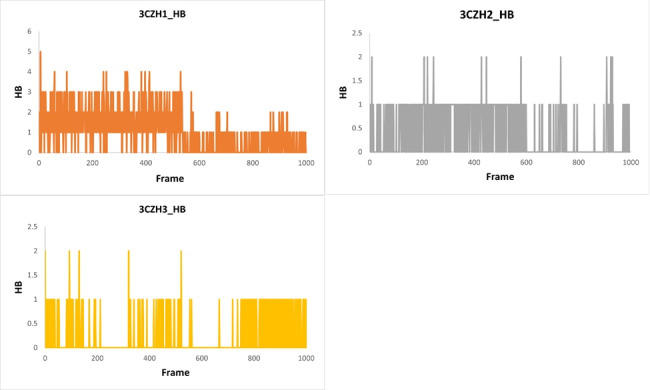
Hydrogen bonding histograms of 3CZH-complex

The antiproliferative effects of *Vallisneria spiralis L*. extracts of AgNPs and IONPs (6.25-100 µg/mL) on MCF-7 BCa cell lines were determined using an MTT assay. The findings demonstrated that increasing the concentration of *Vallisneria spiralis* AgNPs and IONPs resulted in a reduction in the number of viable cells owing to the induction of greater cytotoxicity compared to untreated cells. Among the tested nanoparticles, *Vallisneria spiralis* AgNPs exhibited the highest toxicity towards cancer cell lines. These nanoparticles demonstrated IC50 values of 18.26 µg/mL against MCF-7 BCa cells. In comparison, *Vallisneria spiralis* IONPs showed IC50 values of 42.22 µg/mL when tested against the same MCF-7 BCa cells (Figure 2). The RMSD results showed that proteins mostly deviated between 2 and 2.5 nm. After forming complexes with different compounds, including 3CZH1, 3CZH2 and 3CZH3, the 3CZH1 complex showed greater stability and steady behavior than proteins and other ligands, which validated our docking study. The 3CZH2 complex showed stability, with the highest deviation during the simulation, which was even higher than that of the protein. Compound 3CZH3 stabilizes the protein complex and shows less deviation than the protein with more conformational changes. Overall, 3CZH1 exhibited greater stability and linearity than the others. The comparative RMSD graph is shown in the Figure 5. The resulting RMSF plot indicates that both standalone proteins and protein-ligand complexes exhibit similar patterns of fluctuation, albeit with varying intensities and a few notable exceptions. Protein fluctuation at 120 nm completely disappeared in all complexes. Similarly, the fluctuations between residues 200 and 300 changed in different ways in different complexes. Another prominent change was observed at 380-450 residues. The RMSF graph is shown in Figure 6. To validate the docking outcomes, inhibitory effectiveness and hydrogen bond frequency during molecular dynamics (MD) simulation, an examination of hydrogen bond interactions was conducted. The findings indicated that among the compounds studied, 3CZH1 exhibited the highest hydrogen bonds throughout the molecular dynamics simulation process. The highest number of hydrogen bonds was five, while the lowest number was one, with an average of 3. The other ligands, 3CZH2 and 3CZH3, mostly interacted through single hydrogen bonds throughout the simulation. Figure 7 shows hydrogen-bonding histograms of the ligands.

**Figure 8:**
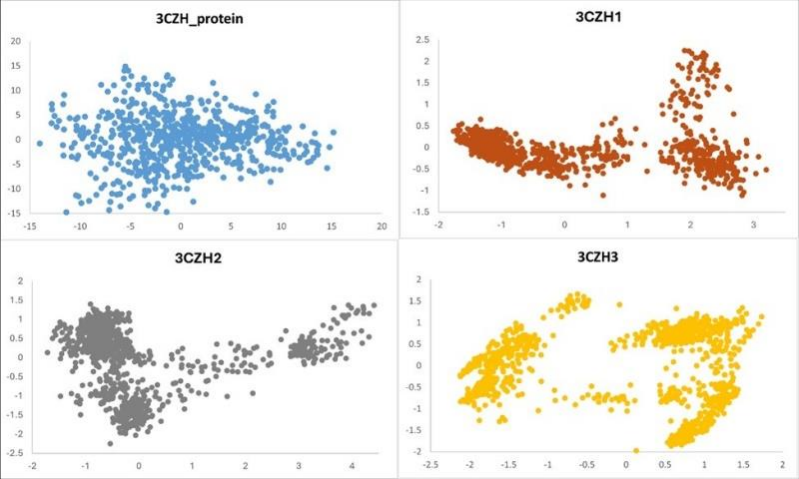
PCA - 2D plot of 3CZH and 3CZH-complex

The GROMACS software was utilized to analyze all proteins and their complexes with ligands. The PCA results revealed that proteins exhibit a wide range of conformational possibilities prior to ligand binding. Upon forming complexes with ligands, the proteins' conformational space becomes more restricted, while the resulting complexes maintain stability. There are only three conformational states in all the complexes: 3CZH1, 3CZH2 and 3CZH3. Figure 8 includes the PCA results for the protein and its variants 3CZH1, 3CZH2 and 3CZH3.

**Figure 9:**
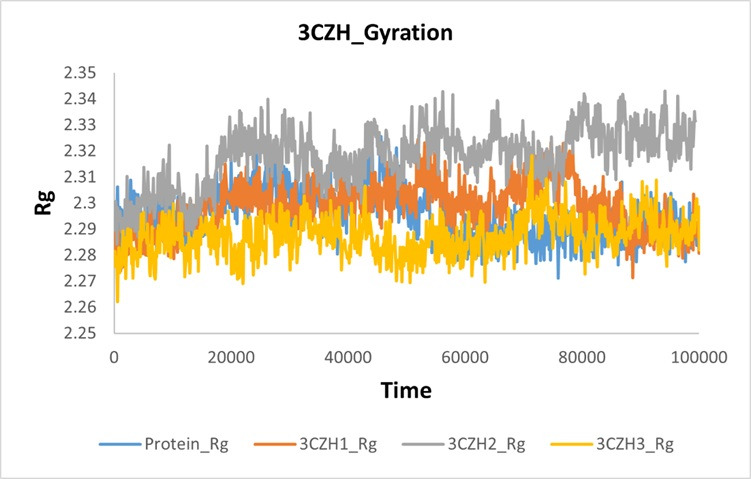
Gyration plot of 3CZH and 3CZH-complex

The compactness of proteins during MD simulation was examined using a gyration plot. Findings indicated that when 3CZH2 was bound to the protein, its compactness decreased, resulting in an increase in size. In contrast, the complexation of 3CZH1 and 3CZH3 with the target protein 3CZH led to enhanced compactness (reduced size) and diminished fluctuations. A comparative gyration plot depicting these results is presented in Figure 9.

## Conclusion:

The potential use of *Vallisneria spiralis Linnaeus* as a natural alternative in breast cancer treatment is shown. The findings demonstrated that increasing the concentration of *Vallisneria spiralis* AgNPs and IONPs resulted in a reduction in the number of viable cells owing to the induction of greater cytotoxicity compared to untreated cells. The biosynthesized Nano particulate drug delivery system of *Vallisneria spiralis* demonstrated antioxidant, cytotoxic and apoptotic properties.

## Authors' contribution:

R.W. and S.K.P. contributed to the conceptualization of the study and proposed the methodology. R.W. carried out the formal analysis and was involved in the investigation, data curation and writing the original draft preparation. S.K.P. took part in writing the review, editing, supervision and project administration. All authors have read and agreed to the published version of the manuscript.

## Data availability:

The data and supportive information are available in the article.

## Figures and Tables

**Table 1:** Predicted anticancer activity of Vallisneria phytocompounds using Way2Drug (PASS webserver)

**Compound no.**	**Name**	**Molecular Formula**	**Pa**	**Pi**
1	[4-(hydroxymethyl)-2,2,5-trimethylhexan-3-yl] 2-methylpropanoate	C14H28O3	0.122	0.07
2	(3-hydroxy-2,4,4-trimethylpentyl) 2-methylpropanoate	C12H24O3	0.729	0.016
3	4-(2,2,6-trimethyl-7-oxabicyclo[4.1.0]heptan-1-yl)but-3-en-2-one	C13H20O2	0.758	0.014

**Table 2:** ADME properties of *Vallisneria spiralis* Linnaeus phytocompounds predicted by SwissADME

**Swiss ADME**	**Drug likeness Rules**	**Lipinski's rule of 5**									
Phytochemicals	Water Solubility	GI Absorption	LOGP	Hydrogen bond donor	Hydrogen bond acceptor	TPSA (Å2)	MW	Ghose	Veber	Egan	Muegge
[4-(hydroxymethyl)-2,2,5-trimethylhexan-3-yl] 2-methylpropanoate	Soluble	High	3.21	1	3	46.53	244.4	Yes	Yes	Yes	Yes
(3-hydroxy-2,4,4-trimethylpentyl) 2-methylpropanoate	Soluble	High	2.84	1	3	46.53	216.3	Yes	Yes	Yes	Yes
4-(2,2,6-trimethyl-7-oxabicyclo[4.1.0] heptan-1-yl)but-3-en-2-one	Soluble	High	2.83	0	2	29.6	208.3	Yes	Yes	Yes	Yes
2,2,6-trimethylcyclohexane-1, 4-dione	Soluble	High	1.63	0	2	34.14	154.2	No	Yes	Yes	No
Note. From *In Silico* and
*In Vitro* Evaluation of Bioactive Compounds of
*Vallisneria spiralis L*. against Candida albicans, by
Waykar R & Kumarapillai S. Open Med Chem J. 2024 18:e18741045314049.
[DOI: 10.2174/0118741045314049240904163514].

**Table 3:** Toxicity prediction of *Vallisneria spiralis Linnaeus* phytocompounds predicted by admetSAR and PROTOX-II software

	**Protox II**			**admet SAR**		
Phytochemicals	LD50 mg/kg	Hepatotoxicity	Mutagenicity	AMES Toxicity	Tetrahymena Pyriformis Toxicity pIGC50 ug/L	Rat Acute Toxicity LD50 mol/kg
[4-(hydroxymethyl)-2,2,5-trimethylhexan-3-yl] 2-methylpropanoate	8350	No	No	No	-0.2728	1.7762
(3-hydroxy-2,4,4-trimethylpentyl) 2-methylpropanoate	3200	No	No	No	-0.2129	1.799
4-(2,2,6-trimethyl-7-oxabicyclo[4.1.0]heptan-1-yl)but-3-en-2-one	2000	No	No	No	0.547	1.788
2,2,6-trimethylcyclohexane-1,4-dione	2400	No	No	No	0.523	2.3921
Note. From *In Silico* and
*In Vitro* Evaluation of Bioactive Compounds of
*Vallisneria spiralis L*. against Candida albicans, by
Waykar R & Kumarapillai S. Open Med Chem J. 2024 18:e18741045314049.
[DOI: 10.2174/0118741045314049240904163514].

**Table 4:** Molecular docking experiments between the protein (3CZH) and its ligands

**Compound No.**	**Chemical name**	**Docking Compound Code**	**Molecular Formula**	**PDB ID**	**Binding energy**
1	[4-(hydroxymethyl)-2,2,5-trimethylhexan-3-yl] 2-methylpropanoate	3CZH1	C14H28O3	3CZH	-6.08
2	(3-hydroxy-2,4,4-trimethylpentyl) 2-methylpropanoate	3CZH2	C12H24O3	3CZH	-4.84
3	4-(2,2,6-trimethyl-7-oxabicyclo[4.1.0] heptan-1-yl)but-3-en-2-one	3CZH3	C13H20O2	3CZH	-7.82
